# Visible Light-Cured Antibacterial Collagen Hydrogel Containing Water-Solubilized Triclosan for Improved Wound Healing

**DOI:** 10.3390/ma14092270

**Published:** 2021-04-27

**Authors:** Longhao Jin, Kyeongsoon Park, Yihyun Yoon, Hyeon Soo Kim, Hyeon Ji Kim, Jae Won Choi, Deuk Yong Lee, Heung Jae Chun, Dae Hyeok Yang

**Affiliations:** 1 Department of Orthopedic Surgery, Yanbian University Hospital, Yanji 133000, China; jlh0423@sina.com; 2 Department of Systems Biotechnology, Chung-Ang University, Anseong 17546, Gyeonggi, Korea; kspark1223@cau.ac.kr (K.P.); kk07gg@cau.ac.kr (H.J.K.); 3 Institute of Cell and Tissue Engineering, College of Medicine, The Catholic University of Korea, Seoul 06591, Korea; dosk9997@catholic.ac.kr (Y.Y.); 10ksu@catholic.ac.kr (H.S.K.); chunhj@catholic.ac.kr (H.J.C.); 4 Lumenbio Co. Ltd., Seoul 08590, Korea; aa@lumenbio.co.kr; 5 Department of Biomedical Engineering, Daelim University, Anyang 13916, Gyeonggi, Korea; dylee@daelim.ac.kr; 6 Department of Biomedical & Health Sciences, College of Medicine, The Catholic University of Korea, Seoul 06591, Korea

**Keywords:** methacrylated collagen hydrogel, 2-hydroxypropyl-beta-cyclodextrin, triclosan, inclusion complex, antibacterial wound dressing

## Abstract

Infection is one of several factors that can delay normal wound healing. Antibacterial wound dressings can therefore promote normal wound healing. In this study, we prepared an antibacterial wound dressing, consisting of visible light-cured methacrylated collagen (ColMA) hydrogel and a 2-hydroxypropyl-beta-cyclodextrin (HP-β-CD)/triclosan (TCS) complex (CD-ic-TCS), and evaluated its wound healing effects in vivo. The ^1^H NMR spectra of ColMA and CD-ic-TCS revealed characteristic peaks at 1.73, 5.55, 5.94, 6.43, 6.64, 6.84, 6.95, 7.31, and 7.55 ppm, indicating successful preparation of the two material types. In addition, ultraviolet–visible (UV–vis) spectroscopy proved an inclusion complex formation between HP-β-CD and TCS, judging by a unique peak observed at 280 cm^−1^. Furthermore, ColMA/CD-ic-TCS exhibited an interconnected porous structure, controlled release of TCS, good biocompatibility, and antibacterial activity. By in vivo animal testing, we found that ColMA/CD-ic-TCS had a superior wound healing capacity, compared to the other hydrocolloids evaluated, due to synergistic interaction between ColMA and CD-ic-TCS. Together, our findings indicate that ColMA/CD-ic-TCS has a clinical potential as an antibacterial wound dressing.

## 1. Introduction

Wound dressings are widely used to protect the wound bed and accelerate wound healing [1]. For optimal wound healing, the ideal wound dressing should be non-toxic, have suitable viscosity and mechanical properties, and be able to absorb exudate, prevent bacterial infections, and maintain humidity around the wound [2].

Hydrogels have many of the properties listed above and can therefore accelerate wound healing [3]. The three-dimensional structure of hydrogels enables them to retain water molecules and thus to provide a moist wound bed environment. This allows for proper cell adhesion and proliferation and thereby accelerates wound healing [4,5]. In addition, the optimal hydration of hydrogels around a wound induces improved autolytic debridement, angiogenesis, and collagen formation [6]. The extent of hydration in hydrogel matrix was characterized by several analytical methods, such as high-resolution magic angle spinning nuclear magnetic resonance (HRMAS-NMR), Fourier-transform infrared attenuated total reflectance (FTIR-ATR) spectroscopy, and Raman spectroscopy [7,8]. Hydrogels, however, also have some disadvantageous mechanical and pharmacokinetic properties. In addition, they lack affinity for cells, which is needed for wound healing [9,10]. Collagen is a good wound dressing material as it possesses good biocompatibility, biodegradability, and cytocompatibility [11,12]. However, collagen hydrogels formed by self-assembly are not suitable for soft tissue regeneration due to the low flexibility of the polymer [13]. Various types of collagen hydrogels prepared by physicochemical crosslinking have been introduced, but the relatively high toxicity of the crosslinkers used has limited their biomedical applications [14].

**Figure 1:**
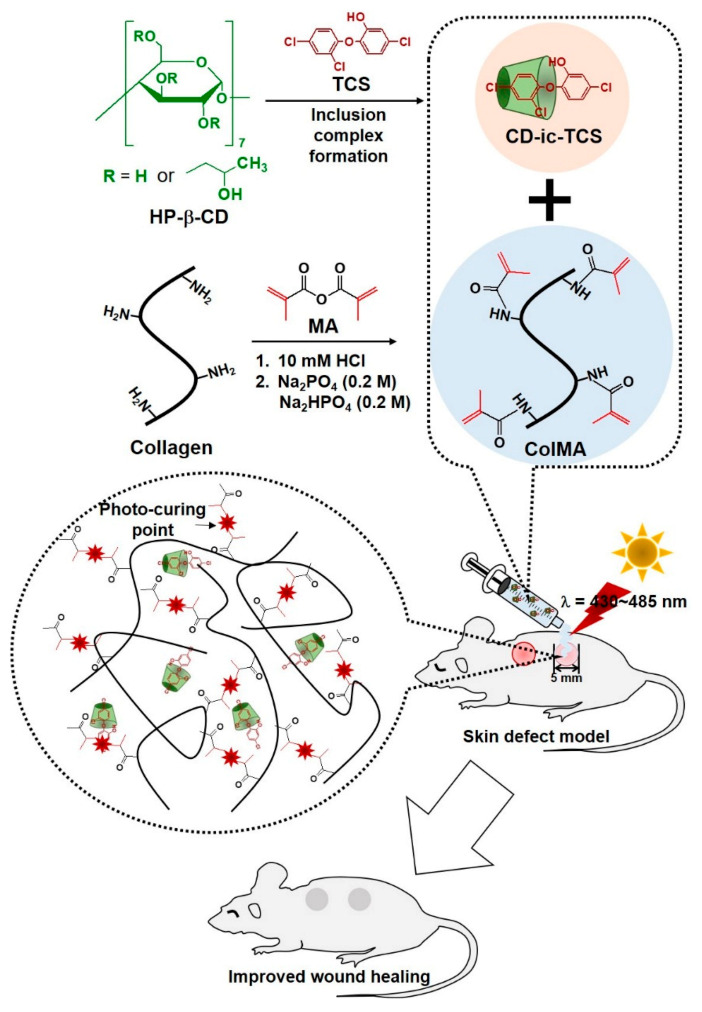
Schematic illustration of the preparation of CD-ic-TCS and ColMA, and the in vivo animal test of ColMA/CD-ic-TCS using a skin defect model. CD-ic-TCS—inclusion complex between 2-hydroxypropyl-beta cyclodextrin (HP-β-CD) and triclosan (TCS); ColMA—methacrylated collagen formed by conjugation of methacrylic anhydride (MA) to a collagen backbone; ColMA/CD-ic-TCS—CD-ic-TCS-incorporated ColMA hydrogel prepared by visible light irradiation (λ = 430–485 nm).

Photopolymerization using visible or ultraviolet light is an attractive method for preparing collagen hydrogels with appropriate mechanical properties, such as photo-curing time [15,16,17,18,19]. Therefore, the mechanical strength of the resulting hydrogel, which affects the pharmacokinetic release parameters of drugs/proteins incorporated in the hydrogel, can be controlled [20]. Visible light is safer to use than ultraviolet light. In addition to visible light-cured hydrogel systems, antimicrobial agents as well are accelerators of wound healing. One of them, triclosan, has a broad-spectrum antimicrobial activity and low toxicity to human skin; however, it has poor water solubility [21,22]. In this study, we evaluated the wound healing efficacy of a novel type of antimicrobial dressing agent based on a visible light-cured methacrylated collagen hydrogel containing a beta-cyclodextrin/triclosan inclusion complex (ColMA/CD-ic-TCS) using a mouse skin defect model (Figure 1). Methacrylated collagen was characterized by proton nuclear magnetic resonance (^1^H NMR). Beta-cyclodextrin (β-CD) was used to improve the water-solubility of triclosan (TCS) because of its ability to promote inclusion complex formation. The inclusion complex between β-CD and triclosan was analyzed by ^1^H NMR and UV spectroscopy. Additionally, we performed in vitro biocompatibility testing and in vivo experiments.

## 2. Materials and Methods

### 2.1. Materials

Collagen was purchased from MSBIO, Inc. (Seongnam, Kyunggi, Korea). Methacrylic anhydride (MA), sodium phosphate monobasic, sodium phosphate dibasic, 2-hydroxypropyl-beta-cyclodextrin (HP-β-CD), and triclosan (TCS) were obtained from Sigma-Aldrich (St. Louis, MO, USA). Riboflavin 5′-monophosphate sodium salt (riboflavin) as a photoinitiator was obtained from Santa Cruz (Santa Cruz, CA, USA). Dialysis membrane tubes (cut-off: 25 kDa and 3.5–5.0 kDa; Spectrum Laboratories Inc., Rancho Dominguez, CA, USA) were used for methacrylated collagen (ColMA). The NIH3T3 mouse fibroblast cell line was obtained from the Korean Cell Line Bank (Seoul, Korea).

### 2.2. Preparation of Methacrylated Collagen (ColMA) Derivative

ColMA was prepared in three steps as follows [23]. First, collagen (1 g) was dissolved in 10 mM HCl solution (300 mL). Then, methacrylic anhydride (MA, 0.5 g) was added to the acidic collagen solution, and the reaction then proceeded at 4 °C for 16 h. The reactant was dialyzed in a 10 mM HCl solution using a dialysis membrane tube (cut-off: 25 kDa) for 2 days. Before use, the purified ColMA solution was lyophilized at −90 °C using a lyophilizer (FD8508; IlShinBioBase Co. Ltd., Yangju, Kyunggi, Korea). Third, the acidic ColMA solution was neutralized using two solutions, namely 0.2 M sodium phosphate monobasic (solution A, NaH_2_PO_4_) and 0.2 M sodium phosphate dibasic (solution B, Na_2_HPO_4_), for dissolution. Solutions A and B were mixed at a 19:81 ratio (*v/v*%) (solution C) prior to addition of collagen, and all solutions were stored overnight in a refrigerator (4 °C) before use. Afterward, the three solutions were then mixed at 2 to 1 (*v/v*%) or 3 to 1 (*v/v*%) ratios to adjust the pH to 6.4~6.8. Finally, the pH was adjusted to 7.4 using a 1 N NaOH solution (25–65 μL/mL). The final product was analyzed by proton nuclear magnetic resonance (^1^H NMR; Bruker Avance; Bruker Avance 400; Harwell, UK) in the co-solvents of D_2_O and acetic acid-*_d4_*.

### 2.3. Inclusion Complex between 2-Hydroxypropyl-Beta-Cyclodextrin and Triclosan (CD-ic-TCS)

HP-β-CD (1 g) and TCS (100 mg) were dissolved in distilled water (10 mL) and acetone (1 mL), respectively [24]. The TCS solution was slowly dropped in the aqueous HP-β-CD solution with continuous agitation, and then the mixture was continuously stirred at room temperature for 24 h to evaporate the acetone. The complex solution was stored in a refrigerator (2–4 °C) before use. The inclusion complex was analyzed by ^1^H NMR (D_2_O) and ultraviolet–visible (UV–vis) spectroscopy (Multiskan^®^ Spectrum; Thermo Fisher Scientific; Waltham, MA, USA) using quartz cuvettes (frosted wall, 0.7 mL).

### 2.4. Preparation of CD-ic-TCS-Loaded ColMA Hydrogel (ColMA/CD-ic-TCS)

The complex solution was added to the ColMA hydrogel precursor solution [25]. The two solutions were mixed until a homogeneous mixture was obtained and photo-cured using riboflavin (12 μM; DPBS) as a photoinitiator for 40 s, and then stored overnight in a refrigerator (2~4 °C) for stabilization. The mixture was transferred into a 1 mL syringe and stored in a refrigerator (2~4 °C) before use. SEM (S-3000H, Hitachi, Japan) was employed for observing the morphologies of hydrogel samples. Prior to observation, the samples were attached on metal mounts with a carbon tape and coated with Au/Pd.

### 2.5. In Vitro Release Test

The release behavior of TCS from the ColMA/TCS and ColMA/CD-ic-TCS hydrogels was analyzed by ultraviolet–visible spectroscopy (Hitachi U-4100, Mountain View, CA, USA) [25]. The amount of TCS in both hydrogels was 10 mg. The hydrogels were added to dialysis membrane tubes (cut-off: 3.5–5.0 kDa) and immersed in PBS (20 mL; pH 7.4) at 37 °C. At each time interval (1, 3, 6, 12, 24, 48, 72, 96, 120, 144, and 168 h), 2 mL of PBS was extracted from each sample, and the same volume of fresh PBS was added. This test was performed in triplicate. The absorbance of the extracted PBS was measured at 282 nm.

### 2.6. In Vitro Biocompatibility

In vitro cytotoxicity testing to investigate the biocompatibility of ColMA/CD-ic-TCS was performed using NIH3T3 cells (Korean Cell Line Bank, Seoul, Korea) between passages 10 and 15. As a control, cells were seeded on a 24-well plate and cultured in Dulbecco’s Modified Eagle Medium containing 25 mM HEPES, 25 mM NaHCO_3_, and heat inactivated bovine calf serum. Three kinds of hydrogels, DuoDERM^®^, ColMA (TCS: 1.8 μg), and ColMA/TCS (TCS: 1.8 μg), were placed in a 96-well plate. After the seeding of 1 × 10^4^ cells/well on each hydrogel, the cells were incubated for 1, 3, and 7 days in an incubator at 37 °C and 5% CO_2_. At specific time intervals, the cell-seeded hydrogels were treated with CCK-8 (100 μL), and this was followed by a 2 h incubation. After extracting supernatants from the CCK-8-treated wells, the optical densities of the supernatant samples were measured at 450 nm using a microplate reader (SpectraMax^®^ i3; Molecular Devices, Sunnyvale, CA, USA) [25].

### 2.7. Antibacterial Assay

Antibacterial activity was evaluated according to a previous study [25,26]. Five hundred microliters of diluted *S. aureus* and *E. coli* (1 × 10^6^ bacterial/mL) were added to the wells of a 48-well plate containing DuoDERM^®^, ColMA, ColMA/TCS, or ColMA/CD-ic-TCS hydrogels (TCS: 1.8 μg), and the plates were incubated at 37 °C for 2 h. Then, 1 mL of PBS was added to each well to resuspend live bacteria. Aliquots of 100 μL of the suspension from each well were plated on Luria-Bertani agar (LB agar) plates, and these plates were incubated at 37 °C for 24 h. Numbers of bacterial colonies were recorded, and kill percentage was calculated as follows:

Kill% = {(cell count of control-survivor of hydrogels)/cell of control} × 100

### 2.8. In Vivo Animal Testing

Animal experiments were approved by the Institutional Animal Care and Use Committee of Chung-Ang University (13 April 2020; 2020-00037) [21]. Twenty Balb/C mice (DooYeol Biotech.; Seoul, Korea) of average body weight (20 g, *n* = 4) were used to evaluate wound healing efficacy. The mice were divided into five groups: control, commercially available DuoDERM^®^ gel, ColMA, ColMA/TCS, and ColMA/HPCD-ic-TCS. Hair on the back of the mice was removed with an electrical shaver and hair removal cream. Four circular defects (5 mm) on the back of each mouse were produced using biopsy punches. Prior to the sample treatment of the defects, the hydrogel precursor solutions were transferred to 1 mL syringes. The syringes were used to place 100 μL of a hydrogel solution on the defects. This step was followed by photo-curing with blue light irradiation for 10 s. At predetermined time intervals (1, 7, and 10 days), hydrogel-treated defects were extracted, and gross appearance was observed. The size of the remaining wound was measured using Image J software (National Institutes of Health Bethesda, MD, USA). Extracted skin was fixed in 10% formalin solution and evaluated by hematoxylin and eosin (H&E) and Masson’s trichrome staining.

### 2.9. Histological Evaluation

The formalin-fixed skin tissue containing wounds obtained at 1, 7, and 14 days after wound creation was dehydrated using a series of ethanol solutions [21]. The dehydrated tissues were embedded in paraffin and sectioned to a thickness of 3 μm using a microtome (DSC1; Leica, Wetzlar, Germany). Afterward, sectioned slides were stained using H&E and Masson’s trichrome stains. These sections were observed using a slide scanner (Pannoramic MIDI; 3DHISTECH Ltd., Budapest, Hungary) at 2.0× and 7.0× and a panoramic viewer (Version 1.15.3; Pannoramic MIDI; 3DHISTECH Ltd., Budapest, Hungary) program.

### 2.10. Statistical Analysis

In vitro biocompatibility, antibacterial activity, TCS release, and wound size were evaluated three times, and results were expressed as the mean ± standard deviation of these triplicate measurements. One-way analysis of variance (ANOVA), as implemented in SPSS (SPSS Inc., Chicago, IL, USA), was used to assess the statistical significance of differences among groups (* *p* < 0.05).

## 3. Results

### 3.1. ^1^H NMR Spectra of CD-ic-TCS and ColMA

**Figure 2:**
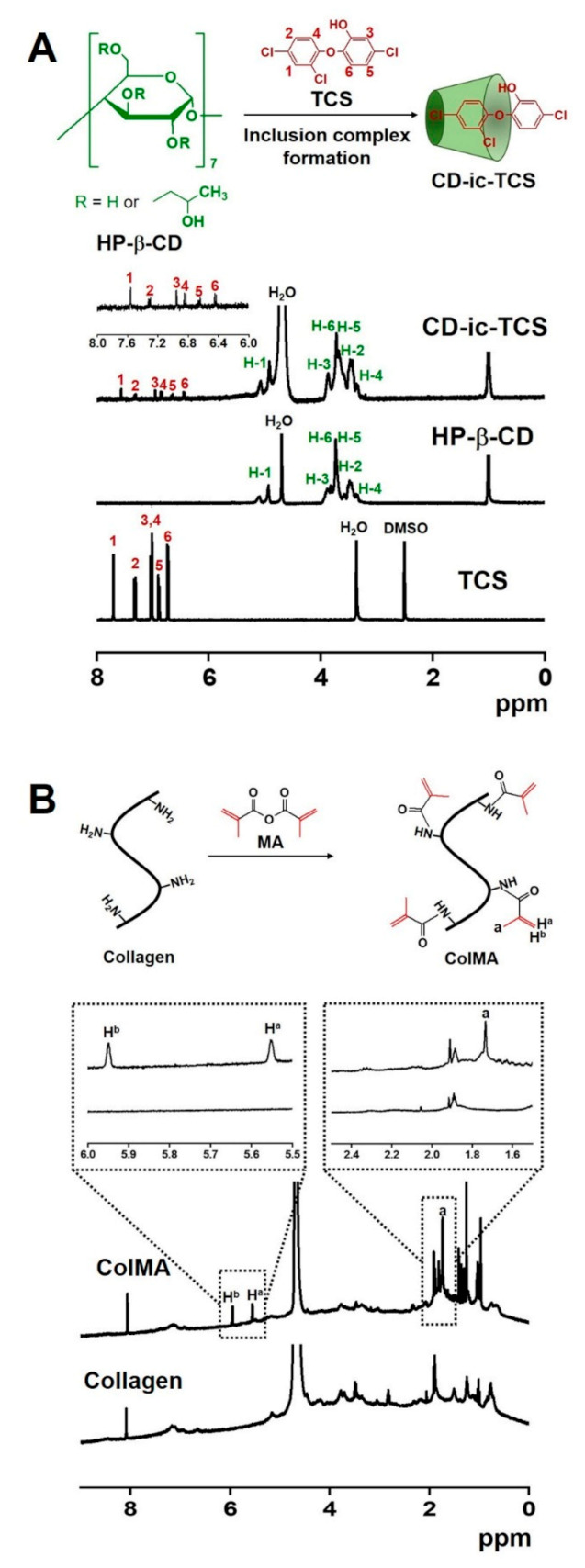
^1^H NMR spectra of (**A**) TCS, HP-β-CD, and CD-ic-TCS; and (**B**) collagen and ColMA. TCS was analyzed using DMSO-*_d6_*. The ^1^H NMR analyses of HP-β-CD and CD-ic-TCS were performed using D_2_O. Collagen and ColMA were analyzed using the co-solvents of D_2_O and acetic acid-*_d4_*.

The inclusion complex between HP-β-CD and TCS and the chemical reaction between collagen and MA were characterized by ^1^H NMR analysis using D_2_O (Figure 2). As shown in Figure 2A, the ^1^H NMR spectrum of CD-ic-TCS was compared to those of HP-β-CD and TCS. HP-β-CD exhibited an H-1 peak at 4.91 ppm and H-2,3,4,5,6 peaks at 3.12–3.98 ppm [27]. In the ^1^H NMR of TCS, six peaks at 6.43, 6.64, 6.84, 6.95, 7.31, and 7.55 ppm were observed [27], corresponding to the ring structure of TCS. CD-ic-TCS had ^1^H NMR characteristics of both HP-β-CD and TCS. We attributed the appearance of the TCS-related peaks in the ^1^H NMR spectrum of CD-ic-TCS to water solubilization of TCS by the inclusion complex formation between HP-β-CD and TCS. The ^1^H NMR spectrum of ColMA is shown in Figure 2B. Compared to the ^1^H NMR spectrum of collagen, new peaks at 1.73, 5.55, and 5.94 ppm were observed for ColMA, which we attributed to the methacrylation containing methyl and vinyl groups. The integration ratio of 5.88 to 5.12 ppm indicated that the substitution degree of methylation was 70%.

### 3.2. UV Spectrum of CD-ic-TCS

**Figure 3:**
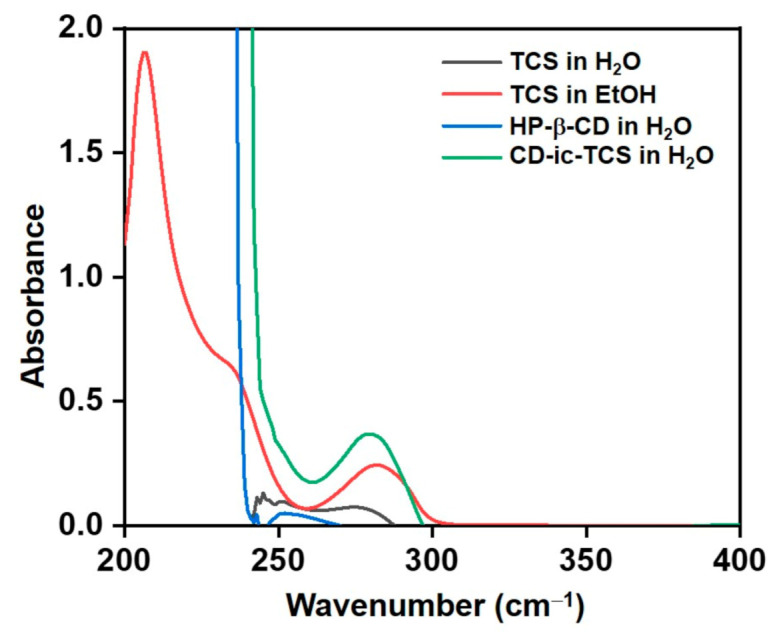
UV spectra of TCS in H_2_O, TCS in EtOH, HP-β-CD in H_2_O, and CD-ic-TCS in H_2_O. The spectra were monitored from 200 to 400 cm^−1^. TCS—triclosan; HP-β-CD—2-hydroxypropyl-beta-cyclodextrin; CD-ic-TCS—inclusion complex between HP-β-CD and TCS.

The inclusion complex formation between HP-β-CD and TCS was further investigated by UV–vis spectroscopy performed in the wavelength range from 200 to 400 cm^−1^ (Figure 3). In aqueous solution, no absorption peak of TCS was detected due to its poor water solubility. By contrast, TCS dissolved in EtOH exhibited an absorption peak at 282 cm^−1^ [28]. No absorption peak of HP-β-CD was found in the monitored wavelength range. CD-ic-TCS showed an absorption peak of TCS at 280 cm^−1^ in an aqueous solution. These results indicated that CD-ic-TCS contributed to the water solubilization of TCS by the inclusion complex formation with HP-β-CD.

### 3.3. SEM Images, TCS Release, and Cell Proliferation

**Figure 4:**
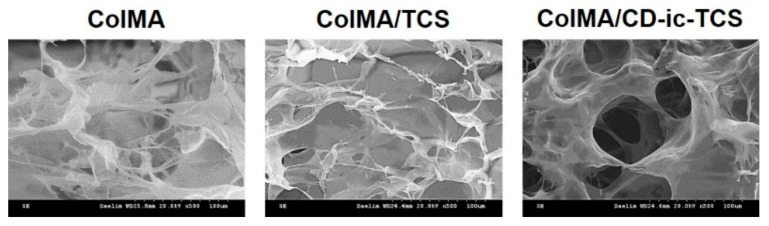
Morphologies of ColMA, ColMA/TCS, or ColMA/CD-ic-TCS observed by SEM at ×500. ColMA—methacrylated collagen; ColMA/TCS—triclosan (TCS) incorporated ColMA; ColMA/CD-ic-TCS—HP-β-CD/TCS complex incorporated ColMA.

**Figure 5:**
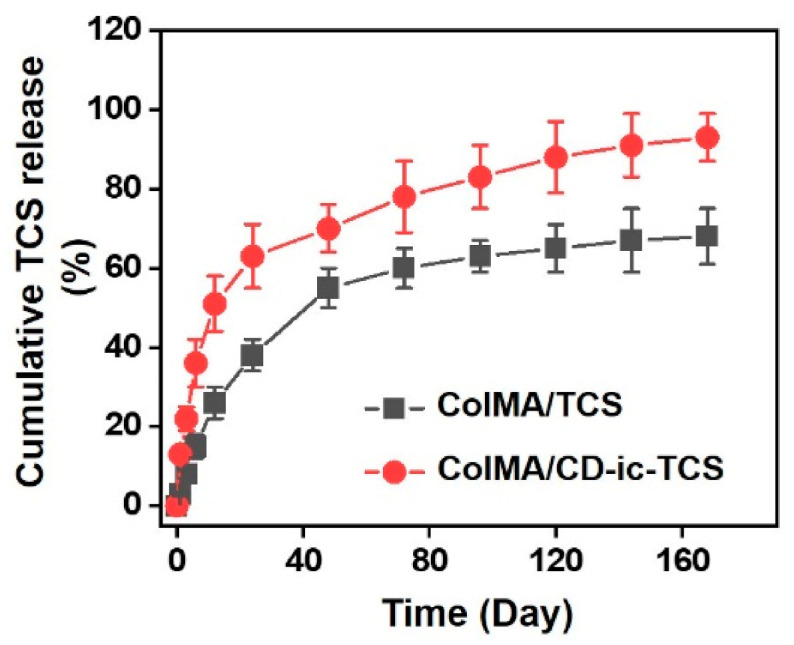
In vitro release behavior of TCS in ColMA/TCS and ColMA/CD-ic-TCS. This test was carried out in triplicate at 37 °C, and the cumulative TCS release percentage was determined at each of the chosen time intervals (1, 3, 6, 12, 24, 48, 72, 96, 120, 144, and 168 h). ColMA/TCS—triclosan (TCS) incorporated ColMA; ColMA/CD-ic-TCS—HP-β-CD/TCS complex incorporated ColMA. This experiment was performed in triplicate (*n* = 3). Results were expressed as mean ± standard deviation.

**Figure 6:**
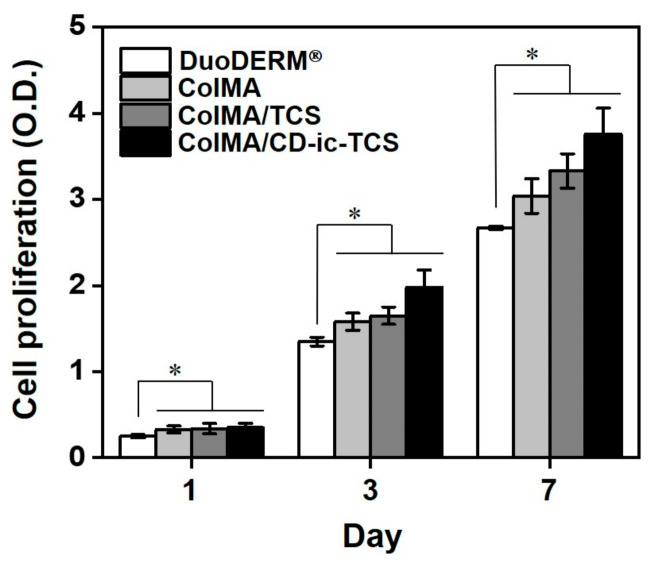
In vitro proliferation of NIH3T3 cells on DuoDERM^®^, ColMA, ColMA/TCS, or ColMA-CD-ic-TCS for culture periods of 1, 3, and 7 days. ColMA—methacrylated collagen; ColMA/TCS—triclosan (TCS) incorporated ColMA; ColMA/CD-ic-TCS—HP-β-CD/TCS complex incorporated ColMA. This experiment was performed in triplicate (*n* = 3). Results were expressed as mean ± standard deviation (* *p* < 0.05).

The microstructural morphologies of ColMA, ColMA/TCS, and ColMA/CD-ic-TCS were investigated by scanning electron microscopy (SEM). Figure 4 shows SEM images of freeze-dried ColMA, ColMA/TCS, and ColMA/CD-ic-TCS observed at ×500. The samples had 3-D networks with interconnected macropores. This may be attributed to the 3-D hydrogel network formation by visible light photo-curing. The interconnected porous structure of hydrogels plays a significant role in their drug release behavior. Figure 5 shows the release behavior of TCS in ColMA/TCS and ColMA/CD-ic-TCS incubated at 37 °C for predetermined time intervals (1, 3, 6, 12, 24, 48, 72, 96, 120, 144, and 168 h). The hydrogels showed two release patterns: an initial burst followed by a controlled release. ColMA/TCS exhibited an initial burst release for 48 h with a controlled release sustained thereafter. Although ColMA/CD-ic-TCS showed a controlled release in a sustained manner along with an initial 24 h burst, it had faster release behavior than ColMA/TCS due to the improved water solubility of TCS by the inclusion complex formation with HP-β-CD. In addition to the drug release behavior, an interconnected porosity provides a more favorable platform for cell proliferation. The proliferation of NIH3T3 cells treated with TCS, ColMA/TCS, or ColMA/CD-ic-TCS for 1, 3, and 7 days is shown in Figure 6. The cell proliferation of all samples increased gradually as a function of culture time. In addition, the ColMA-based samples exhibited a remarkable cell proliferation as compared to DuoDERM^®^ over time. Among ColMA-based samples, ColMA/CD-ic-TCS treatment was associated with the highest cell proliferation. These results indicated that ColMA/CD-ic-TCS had a superior biocompatibility.

### 3.4. Antibacterial Activity

**Figure 7:**
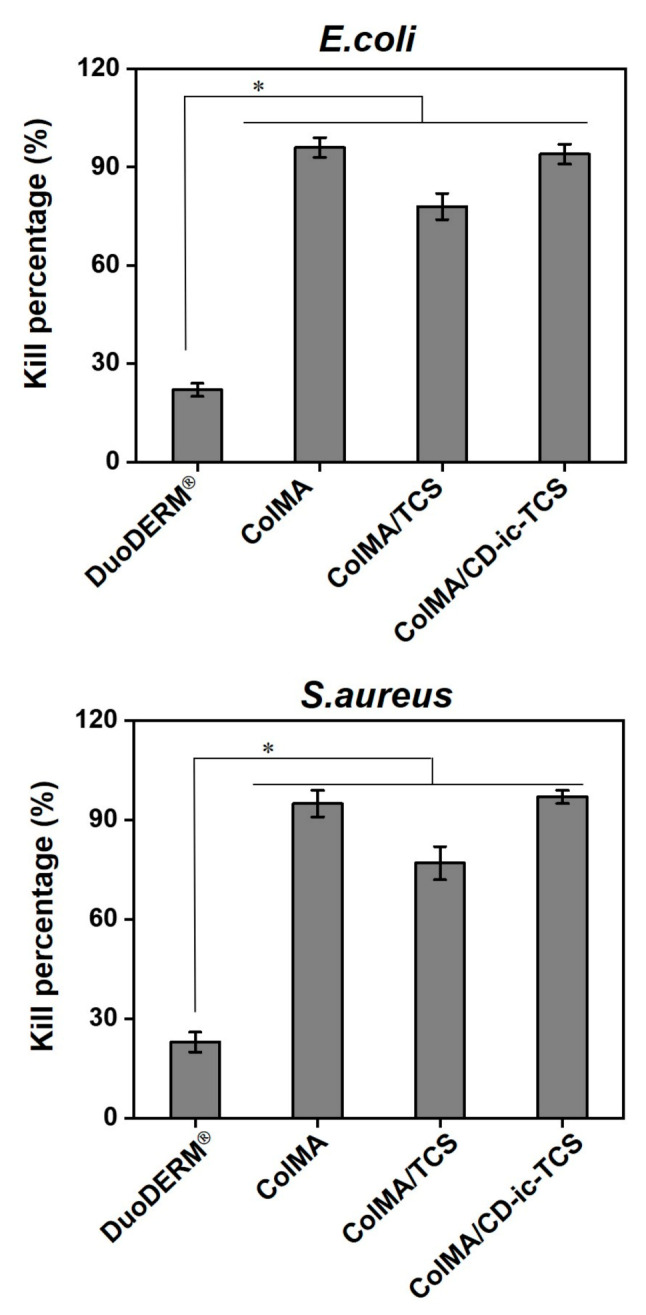
Antibacterial activity of DuoDERM^®^, ColMA, ColMA/TCS, and ColMA-CD-ic-TCS against *E.coli* and *S. aureus*. ColMA—methacrylated collagen; ColMA/TCS—triclosan (TCS) incorporated ColMA; ColMA/CD-ic-TCS—HP-β-CD/TCS complex incorporated ColMA. This experiment was performed in triplicate (*n* = 3). Results were expressed as mean ± standard deviation (* *p* < 0.05).

The antibacterial activity of ColMA/CD-ic-TCS against *E. coli* and *S. aureus* over 48 h was compared to that of DuoDERM^®^, TCS, ColMA, and ColMA/TCS (Figure 7). The samples treated with DuoDERM^®^ or ColMA showed high kill percentages of the bacteria after the 48 h. On the contrary, TCS-related samples had lower kill percentages than DuoDERM^®^ and ColMA. The kill percentage of ColMA/TCS-treated samples remained around 77–78% after 48 h. Moreover, ColMA/CD-ic-TCS-treated samples had the highest kill percentage among all samples, indicated that TCS would increase the kill percentages of *E. coli* and *S. aureus*.

### 3.5. Gross Appearances and Wound Healing

**Figure 8:**
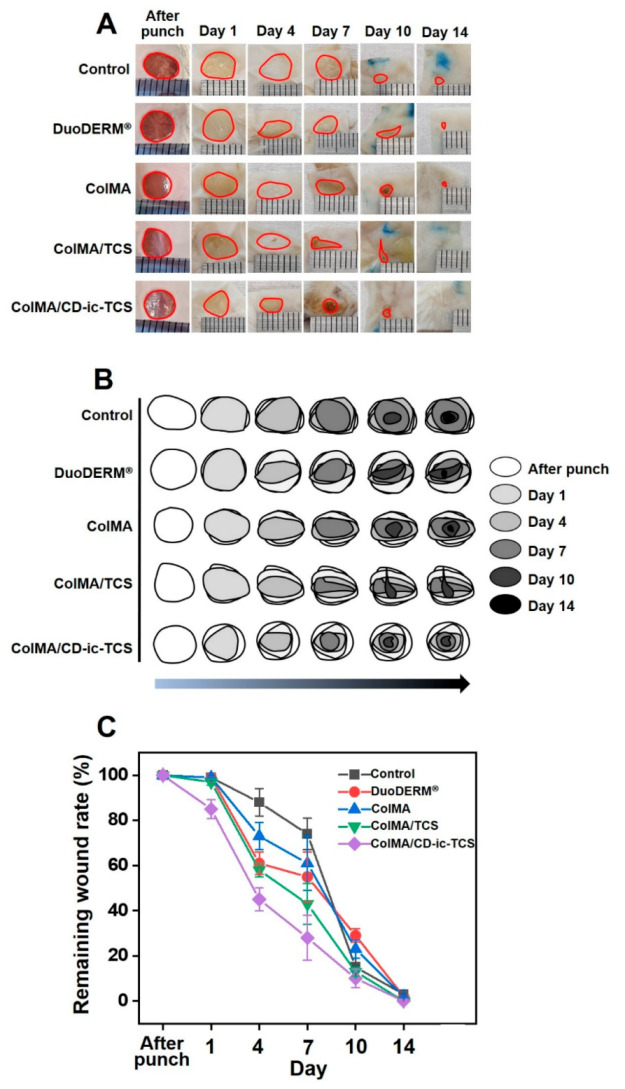
(**A**) Gross appearances of wound defects of control wounds and those treated with DuoDERM^®^, ColMA, ColMA/TCS, or ColMA/CD-ic-TCS for 1, 4, 7, 10, and 14 days. (**B**) Schematic diagram of remaining wound sizes at each time interval. (**C**) Remaining wound rates calculated at each time interval. This experiment was performed three times (*n* = 3). Results were expressed as mean ± standard deviation. ColMA—methacrylated collagen; ColMA/TCS—triclosan (TCS) incorporated ColMA; ColMA/CD-ic-TCS—HP-β-CD/TCS complex incorporated ColMA.

The gross appearance and remaining size of the control and the DuoDERM^®^-, ColMA-, ColMA/TCS-, and ColMA/CD-ic-TCS-treated wounds were investigated after punching, on days 1, 4, 7, 10, and 14 (Figure 8). As shown in Figure 8A, the wound size decreased in all groups over time. Furthermore, compared with the control, DuoDERM^®^, and ColMA samples, full skin-covered wound healing was observed in the ColMA and ColMA/CD-ic-TCS groups. Further examination of wound healing as a function of time was performed (Figure 8B,C). On day 1, mice in the ColMA/CD-ic-TCS group showed more accelerated wound healing than the control mice or the DuoDERM^®^-, ColMA-, or ColMA/TCS-treated mice. On day 3, accelerated wound healing was observed in the hydrogel-treated wounds. In addition, the ColMA/CD-ic-TCS mice showed better wound healing than the DuoDERM^®^, ColMA, and ColMA/TCS mice. Like the results obtained on day 7, the wound healing of the hydrogel-treated mice on day 10 was better than that of the control mice. ColMA/CD-ic-TCS had the greatest wound healing effect among all treatments over 7 days. After day 7, all samples showed similar wound healing. We confirmed that ColMA and the water solubility of TCS accelerated wound healing.

### 3.6. Histological Evaluations

**Figure 9:**
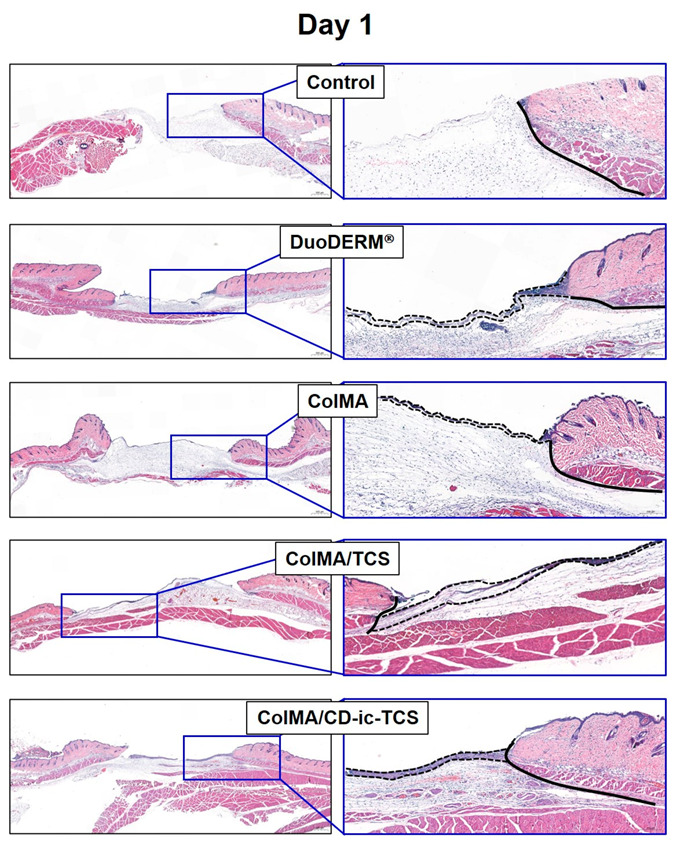

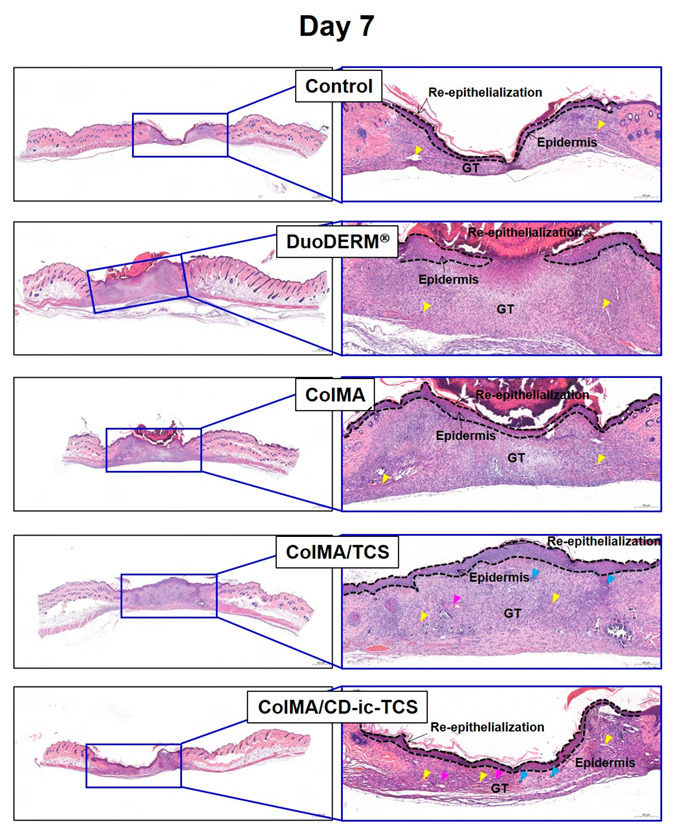

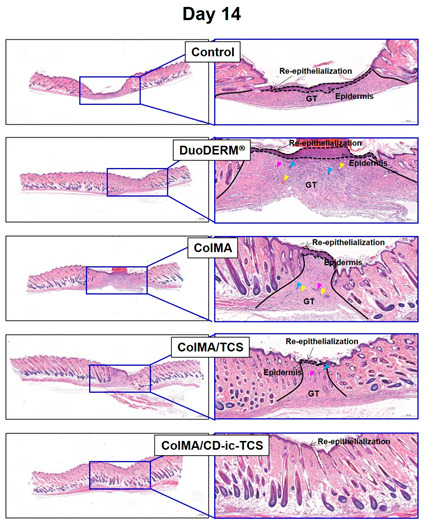
H&E-stained slides of wound defects treated with DuoDERM^®^, ColMA, ColMA/TCS, or ColMA/CD-ic-TCS for 1, 7, and 14 days. The (**left**) and (**right**) images were observed at 2.0× (500 μm) and 7.0× (200 μm), respectively. ColMA/TCS—triclosan (TCS) incorporated ColMA; ColMA/CD-ic-TCS—HP-β-CD/TCS complex incorporated ColMA; GT—granulation tissue. Spaces in black and dotted lines indicate the epidermis and GT, respectively.

Figure 9 shows H&E-stained slides of wound defects treated with DuoDERM^®^, ColMA, ColMA/TCS, or ColMA/CD-ic-TCS, compared to an untreated wound (control). On day 1, inflammatory cells and a provisional extracellular matrix were observed in all groups. Epidermis production began in the wounds of hydrogel-treated groups (DuoDERM^®^, ColMA, ColMA/TCS, and ColMA/CD-ic-TCS). The ColMA/CD-ic-TCS mice showed remarkable epidermis production and angiogenesis, indicating an acceleration of wound healing. On day 7, the inflammatory cells were still observed in all groups. However, all groups also had a fully covered epidermis and a granulation tissue. At this time, compared with the hydrogel-treated groups, the control group exhibited scanty epidermal regeneration in the center of the wound and new blood vessels formation at the wound edges. Similarly to the control, the DuoDERM^®^-treated mice also had wounds fully covered by epidermis and granulation tissue, and angiogenesis around the wound edges was observed. However, the granulation tissue was thicker in the DuoDERM^®^ group than in the control group. The mice treated with ColMA-based hydrogels showed greater wound healing acceleration than those treated with DuoDERM^®^. The ColMA-based hydrogels induced a thinner epithelial layer than DuoDERM^®^. In the case of the hydrogels, ColMA induced angiogenesis around the edges of the wound, whereas angiogenesis was generally observed in the granulation tissues in the ColMA/TCS- and ColMA/CD-ic-TCS-treated groups. Furthermore, the wounds treated with TCS-loaded hydrogels produced hair follicles. On day 14, the wounds treated with ColMA-based hydrogels showed an accelerated wound healing compared to the control and DuoDERM^®^-treated wounds. The hydrogels contributed to the production of hair follicles and sebaceous glands. Moreover, ColMA/CD-ic-TCS promoted wound recovery.

**Figure 10:**
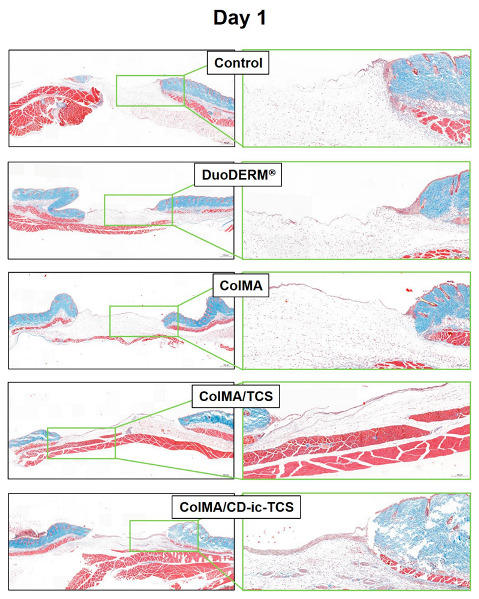

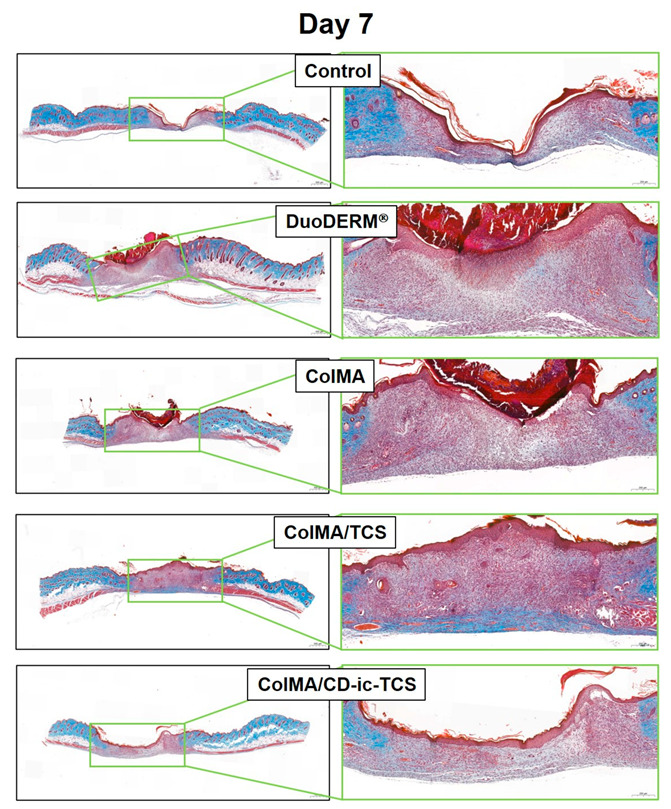

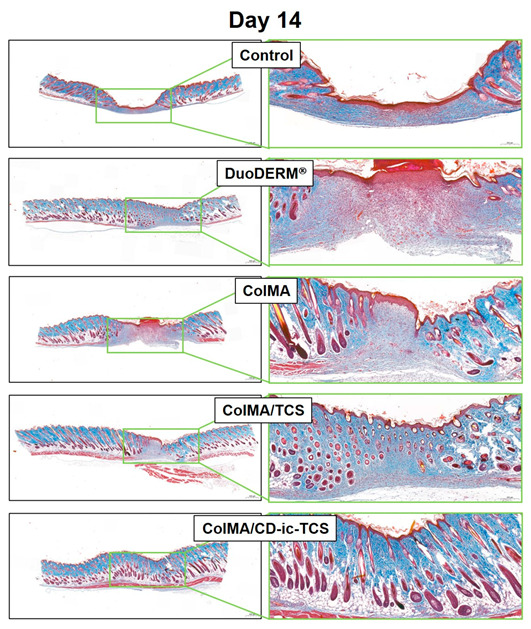
MT-stained slides of wound defects treated with DuoDERM^®^, ColMA, ColMA/TCS, or ColMA/CD-ic-TCS for 1, 7, and 14 days. The left and right images were observed at 2.0× (500 μm) and 7.0× (200 μm), respectively. ColMA/TCS—triclosan (TCS) incorporated ColMA; ColMA/CD-ic-TCS—HP-β-CD/TCS complex incorporated ColMA. Blue color indicates collagen.

Collagen synthesis in the control and hydrogel-treated wounds was investigated by Masson’s trichrome staining (Figure 10). On days 1 and 7, no differences in collagen synthesis were observed among samples. On day 14, collagen synthesis was observed in all samples. Compared with the control and DuoDERM^®^-treated samples, those treated with the ColMA-based hydrogels showed an advanced wound healing. In addition, incorporation of TCS in ColMA further enhanced wound healing. We attributed some of the acceleration of wound healing to the improvement of the water solubility of TCS by inclusion complex formation with HP-β-CD.

## 4. Discussion

The ultimate goal of wound healing is a complete healing of the defect without any lasting effects, such as scar formation. One of the causes of scar formation is bacterial invasion. Natural collagen is widely used in research and clinical practice due to its biocompatibility and low antigenicity. However, natural collagen itself is not appropriate as a wound dressing because it can easily be enzymatically degraded. Several types of collagen-based wound dressings, including hydrogels, sheets, and sponges, have been introduced because the physicochemical properties of the polymer allow for facile remodeling [29]. These collagen-based dressings can advance wound healing by inactivating excessive matrix metalloproteases. In addition, collagen promotes migration of healing-related cells towards the wound, which stimulates angiogenesis, re-epithelialization, and new tissue formation [30].

Among dressing types, hydrogel systems can absorb wound exudates because of their 3-D network. In addition, a hydrogel can be used in wounds of various sizes or shapes because of its ability to swell [3,21,31,32]. Due to its water-insoluble properties, only a few collagen-based hydrogels for wound dressings have been reported [30,33,34]. However, the collagen hydrogels had uncontrollable mechanical properties; on the other hand, the mechanical properties of the photo-cured collagen hydrogel described in this study may be easily controlled by the irradiation time, leading to a custom-made controlled drug delivery system.

In the present study, we developed a collagen-based hydrogel containing TCS as an antibacterial wound dressing (Figure 1). The antibacterial activity of TCS varies according to its concentration. At low concentrations, TCS inhibits the synthesis of fatty acids for of bacterial membranes. At high concentrations, TCS disrupts the bacterial membrane, followed by enhanced antibacterial activity. However, TCS has low bioavailability because of its poor water solubility. We used a photo-curing system to incorporate the TCS into the hydrogel and performed methacrylation of the collagen backbone (ColMA) to prepare a collagen hydrogel (Figure 2) [35]. The inclusion of TCS into β-CD derivatives improved the water solubility of the drug, which was previously confirmed by UV–vis spectroscopy (Figure 3) [36]. Because TCS can be cytotoxic at concentrations greater than 5 μg/mL, we performed in vitro cell-proliferation and antibacterial-activity assays. Our results (Figure 6 and Figure 7) indicated that the ColMA hydrogel was a good material for decreasing the cytotoxicity of TCS and increasing its antibacterial activity [22].

We performed in vivo animal testing to evaluate the feasibility of using ColMA/CD-ic-TCS to accelerate wound healing. The porosity of the collagen-based hydrogel contributed to the controlled release of TCS. Application of β-CD to TCS resulted in inclusion complex formation and faster release of TCS. Both ColMA and CD-ic-TCS improved wound healing, according to our assessment of the gross appearance of wounds and the wound sizes (Figure 6, Figure 7 and Figure 8). Histological evaluations further demonstrated the beneficial effects of the ColMA hydrogel and CD-ic-TCS on wound healing (Figure 9 and Figure 10). After a healing period of 14 days, the mice treated with ColMA/CD-ic-TCS showed complete wound healing. These results suggested that ColMA/CD-ic-TCS promoted wound healing.

## 5. Conclusions

We prepared an antibacterial wound dressing based on a visible light-cured ColMA hydrogel and an HP-β-CD/TCS complex and evaluated its wound healing capacity. The photo-cured ColMA hydrogel and HP-β-CD/TCS were fabricated by photo-curing—using riboflavin as a photoinitiator—and inclusion complex formation, respectively. ColMA/CD-ic-TCS was characterized by ^1^H NMR analysis and UV–vis spectroscopy, which confirmed the successful formation of methacrylated collagen and HP-β-CD/TCS complex, respectively. In addition, in vitro tests, including SEM, a release test, biocompatibility test, and antibacterial assay, showed that the porous ColMA/CD-ic-TCS exhibited a controlled release of TCS, good biocompatibility, and high antibacterial activity. ColMA/CD-ic-TCS also accelerated wound healing in an in vivo mouse model. Together, our findings suggest that ColMA/CD-ic-TCS has the potential for clinical use as an antibacterial wound dressing.

## Data Availability

Not applicable.
